# A new method for selecting sites for soil sampling, coupling global weighted principal component analysis and a cost-constrained conditioned Latin hypercube algorithm

**DOI:** 10.1016/j.mex.2019.02.005

**Published:** 2019-02-08

**Authors:** Kwabena Abrefa Nketia, Stephen Boahen Asabere, Stefan Erasmi, Daniela Sauer

**Affiliations:** a Physical Geography Dept., Georg-August-Universität Göttingen, Germany; b Cartography, GIS & Remote Sensing Dept., Georg-August-Universität Göttingen, Germany; c Council for Scientiﬁc and Industrial Research - Soil Research Institute, Kumasi, Ghana

**Keywords:** Sampling design to represent both the feature and the geographical space, Auxiliary dataset, cLHC, GWPCA, Localised spatial soil variability, Optimised soil sampling design

## Abstract

Analysing spatial patterns of soil properties in a landscape requires a sampling strategy that adequately covers soil toposequences. In this context, we developed a hybrid methodology that couples global weighted principal component analysis (GWPCA) and cost-constrained conditioned Latin hypercube algorithm (cLHC). This methodology produce an optimized sampling stratification by analysing the local variability of the soil property, and the influence of environmental factors. The methodology captures the maximum local variances in the global auxiliary dataset with the GWPCA, and optimizes the selection of representative sampling locations for sampling with the cLHC. The methodology also suppresses the subsampling of auxiliary datasets from areas that are less representative of the soil property of interest. Consequently, the method stratifies the geographical space of interest in order to adequately represent the soil property. We present results on the tested method (*R^2^ = 0.90 and RMSE = 0.18 m*) from the Guinea savannah zone of Ghana.

• It defines the local structure and accounts for localized spatial autocorrelation in explaining variability.
• It suppresses the occurrence of model-selected sampling locations in areas that are less representative of the soil property of interest.

## Graphical abstract

**Figure:**
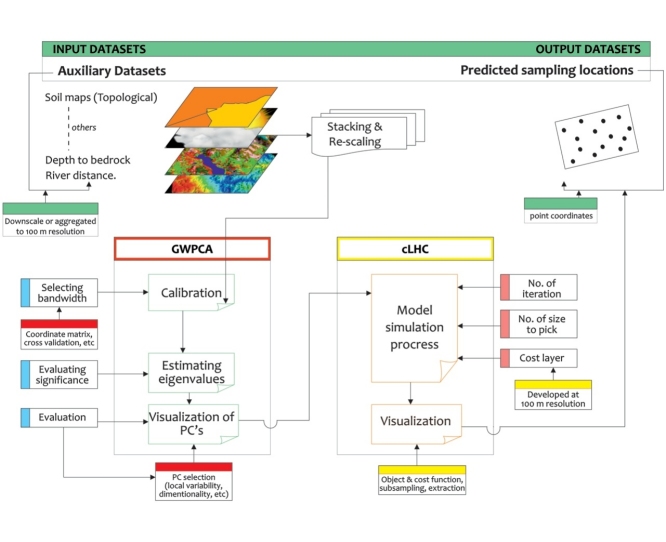

**Specification Table**

**Table:** 

Subject Area	Agricultural and Biological Sciences
More specific subject area:	Soil science
Method name:	Sampling design to represent both the feature and the geographical space
Name and reference of original method	B. Minasny, A.B. McBratney, A conditioned Latin hypercube method for sampling in the presence of ancillary information. Computers & Geosciences 32(9): (2006) 1378–1388.
	P. Roudier, D.E. Beaudette, A.E. Hewitt, A conditioned Latin hypercube sampling algorithm incorporating operational constraints. Digital soil assessments and beyond (2012) 227–231.
	P. Harris, A. Clarke, S. Juggins, C. Brunsdon, M. Charlton, Enhancements to a Geographically Weighted Principal Component Analysis in the Context of an Application to an Environmental Data Set. Geographical Analysis 47(2): (2015) 146–172.
	S. Kumar, R. Lal, C.D. Lloyd, Assessing spatial variability in soil characteristics with geographically weighted principal components analysis. Computational Geosciences 16(3): (2012) 827–835.
Resource availability	Source codes (R scripts) for full method implementation on GitHub repository (https://github.com/kanj241/PhD/blob/master/Sampling_desi-gn).

## Method details

Sampling designs aim at representing either the investigated soil property or the geographical space of a study area, or both [1,2]. However, there are still shortcomings in the geographical-space sampling designs [3]. Minasny and McBratney [2] proposed the conditioned Latin hypercube (cLHC) method as a feature-sampling approach, to address the shortcomings of the geographical-space sampling design. To ensure that a sampling strategy adequately represents both the geographical and the feature space, Minasny and McBratney [2] recommended considering the whole range of auxiliary data available for a study area. Based on this suggestion, Levi and Rasmussen [4] proposed a hybrid approach, in which they coupled an iterative principal component analysis (iPCA) with the cLHC. However, the iPCA hardly accounted for local spatial effects in their auxiliary datasets. The iPCA algorithm generally obscured the localized spatial effects in the auxiliary datasets [[5], [6], [7]]. Generally, the iPCA algorithm is unable to correlate the measured soil property and its local environment, which depicts the geographic variations in the soil and environmental characteristics across space. Hence, iPCA ignores spatial characteristics. Therefore, several authors suggested to correct this shortcoming by including a localized weighted spatial auto-correlated principal component analysis [5,6,8]. However, even coupling iPCA with cLHC still does not account for geographical weightings that provide principal component scores and loadings at all data locations [7]. Therefore, in this paper we propose a global weighted principal component analysis (GWPCA) as an alternative to the iPCA. The advantage of the GWPCA is that it is able to recover the known dimensional spatial structures. Hence, it accounts for localized spatial autocorrelations in the algorithm that can explain the variability of auxiliary datasets [9]. Consequently, we propose a new method, in which we couple GWPCA and a cost-constrained cLHC, to optimize the representation of both the feature and the geographical space.

Similar to the *scorpan* concept [10], auxiliary datasets, represented as indices, were used to explain the local spatial heterogeneities and the soil property of interest at the selected sampling locations. We evaluated all localized spatial effects, trends and variabilities in the auxiliary datasets by GWPCA, adopting an automatic bandwidth in the GWPCA calibration. Next, using the selected GWPCA principal components as model input parameters, we selected optimal sampling locations using the cLHC algorithm executing 1 * 10^4^ — 5 * 10^4^ iterations. We incorporated a cost layer in the cLHC algorithm to suppress subsampling from areas that had only minor influence on the soil variable. Finally, we evaluated the model selections using root mean square error and correlation coefficient between model selected and actual locations. We chose soil moisture (SM) to test the method. In all stages of the proposed hybrid approach, we used R software [11], specifically the R packages *factoextra* [12], *rsaga* [13], *stats* [11], *psych* [14] and *clhs* [15].

### Global weighted principal component analysis (GWPCA)

The GWPCA is an add-on to the standard principal component analysis [[5], [6], [7]]. We evaluated all localized spatial effects, trends and variabilities in the auxiliary datasets by the GWPCA. Geographical weights (GW) used in the GWPCA were determined by a bi-square function (Eq. (1)).

(1) $${GW}_{ij}={\left[{1\;-\left(\frac{d_{ij}}{b}\right)}^{2}\right]}^{2}$$

where $d_{ij}$ is the distance between the spatial location *i* and *j* at a bandwidth *b* in determining the kernel size of the PCA.

Finally, the global weighted principal components at each location *(x_i_, y_i_)* were estimated by Eq. (2).

(2) $${LVL}^{T}|\;\left(x_{i},\;y_{i}\right)=\;\sum \left(x_{i},\;y_{i}\right)$$

where LVL is the local eigenstructure, and $\sum \left(x_{i},\;y_{i}\right)$ is the GW variance-covariance matrix for location $\left(x_{i},\;y_{i}\right)$.

We used a component matrix of the loadings to explore the local variations in the auxiliary data used in this study. Positive/negative signs associated with the loadings indicate, how each auxiliary data is associated with other auxiliary data. The geographically weighted standard deviation of auxiliary datasets was estimated by Eq. (3).

(3) $$\sqrt{\sigma_{i}}={\left[\sum_{j=1}^{n}{\left(x_{1}-\;{\bar{x}}_{1}\right)}^{2}{GW}_{ij}\right]}^{0.5}$$

where $x_{1}-\;{\bar{x}}_{1}$ is the auxiliary data and its mean and ${GW}_{ij}$ is the geographical weights between the spatial location *i* and *j*.

### Cost-constrained conditioned Latin hypercube algorithm (cLHC)

A cost layer was introduced into the simulated annealing process within the cLHC algorithm [15] as Eq. (4). The cost layer suppressed the subsampling of selected PCs of the GWPCA auxiliary data from areas that had only minor influence on SM in the cLHC algorithm outputs.

(4) $$C_{{cost}_{\left(j\right)}}=\;e^{\left(\;-\;\frac{\Delta {cost}_{j}}{T}\right)}$$

where $\Delta {cost}_{\left(j\right)}=cost\left(j\right)-cost(j-1)$. Within the sampling schemes, $C_{{cost}_{\left(j\right)}}$ was the sum of the cost layers of individual locations at *j* iterations in the simulated annealing. Details on applying the standard and cost-constrained cLHC algorithms can be obtained from the studies of Minasny and McBratney [2] and Roudier et al [15].

### Evaluation of the accuracy of the model selected sampling locations in the field

We tested the performance of the hybrid approach in the field, using six covariates, including soil type, parent material, landform, drainage, effective soil thickness, and the possibility to fix access tubes without impedance (Table 1). The selection of these indicators was corroborated by studies of Adu [16] and Adu and Asiamah [17] in the Guinea savannah zone. We evaluated each model selected sampling location in the field, by assigning either a value of 0 (= unsuitable) or 1 (= suitable) to each of the six parameters. Subsequently, the total score of each selected sampling location was averaged and expressed as percentage. For the selected sampling locations, the root mean square error (RMSE) and the correlation between the predicted and actual location was estimated. In this way, we evaluated the average error and the suitability of the model to select representative sampling locations (Eq. (5)).

(5) $$RMSE=\;\sqrt{\frac{1}{n}\sum_{i=1}^{n}{\left[P_{t}-\;A_{t}\right]}^{2}}$$

where *P* is the model selected sampling location at a feature space *t*, *n* is the number of model selected sampling locations and *A* is the confirmed/actual *in-situ* sampling location.

**Table 1:** Evaluation form to confirm the suitability of predicted sampling location.

Locations	Field Conditions						
	Soil type	Geology	Land form	Possibility to fix access tube	Drainage	Effective soil depth > 100 cm	0|1 Score
AT01							
AT02							
…							
…							
…							
AT38							

## Validation of the proposed hybrid methodology

### Characteristics of the study area where the methodology was tested

**Fig. 1:**
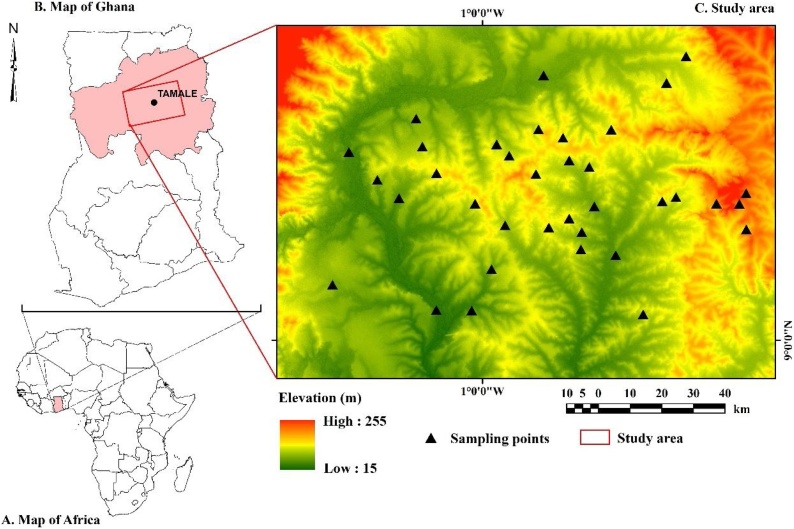
Maps showing the locations of the study area (A, B) and sampling sites, super-imposed on the digital elevation model (C).

The study area (Fig. 1) is a major agricultural area of Ghana. Agriculture is the main base of the economic livelihood of the local population. The area is semi-arid, characterized by a single rainfall season, peaking in June, and a mean annual rainfall of 1200 mm. The daily mean temperatures vary between ˜32 °C (in August) and ˜43 °C (in March), and mean daily relative humidity varies between 18% and 97%. Elevation ranges from ˜39 m to ˜255 masl. The study area is largely flat with gentle slopes of 0.5–5% inclination.

**Fig. 2:**
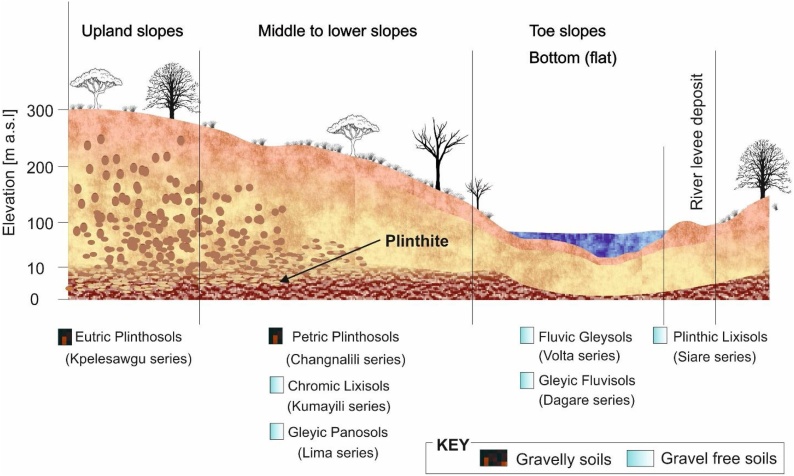
Soil types along the toposequence. Soil names are in Ghanaian local classification system. Chart not drawn to scale.

The study area has seven benchmark soils, reported here according to the World Reference Base for Soil Resources [18]. The soils developed mainly on sandstone, shale, mudstone and quartzite of the Voltain platform, and alluvial sediments [19]. Three main topographical units can be distinguished in the study area, *i.e*. upper slopes, middle to lower slopes, and toe slopes. The upper slopes are widely covered by Eutric Plinthosols (Kpelesawgu series, in the local classification system). On the middle to lower slopes, Petric Plinthosols (Changnalili series), Chromic Lixisols (Kumayili series) and Gleyic Panosols (Lima series) occur. The soil association on the toe slopes includes Fluvic Gleysols (Volta series), Plinthic Lixisols (Siare series) and Gleyic Fluvisols (Dagare series) (Fig. 2). Details on these soils were reported by Adu [16] and Adu and Asiamah [17]. The vegetation of the area is mainly grassland with interspersed shea trees (*Vitellaria paradoxa*), Borassus palm (*Borassus aethiopum*) and Senegal mahogany (*Khaya senegalensis*).

### Auxiliary datasets used in the study

**Fig. 3:**
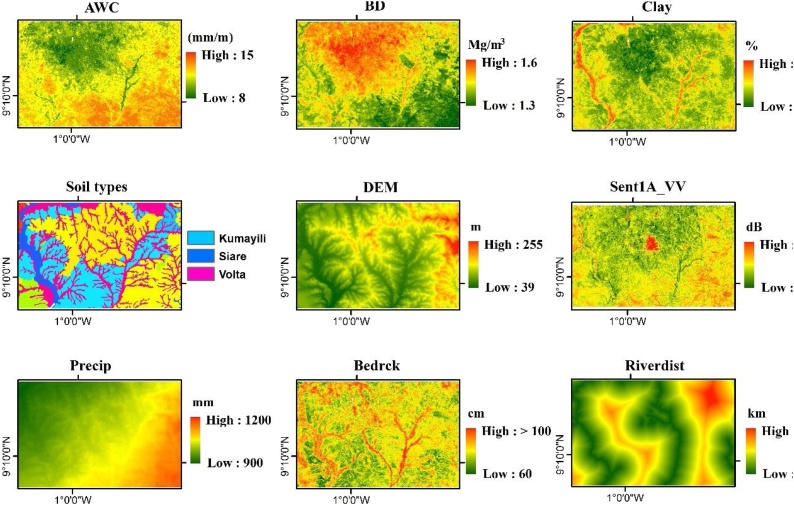
Extract from list of auxiliary datasets used to analyze the soil-landscape heterogeneity in representing SM. AWC = available water content, BD = bulk density, Clay = clay content, Sent1A_VV = calibrated Sentinel-1 radar backscatter coefficient in VV polarization, Precip = precipitation, Bedrck = depth to bedrock, Riverdist = river distance.

We selected twenty auxiliary datasets, which we considered to represent factors that contribute to the local spatial variability of SM. We thus expected these datasets to be suitable to explain the SM dynamics at the model selected sampling locations. They were also included in the modelling process, in order to optimize the selection of sampling locations. Collinearity between the auxiliary datasets was accounted for with the GWPCA algorithm [5]. Table 2 and Fig. 3 present summaries of the auxiliary datasets used in this study. Each auxiliary dataset was either sourced or resampled at 100 m resolution via the cubic spline model of the *R-GDAL* package [20]. DEM-derived surfaces were obtained via the *SAGA-ta_morphometry* and *SAGA-ta_hydrology* functions [21].

**Table 2:** Auxiliary datasets used in the study.

Abbreviation	Description	Remarks	Units
DEM	Digital elevation model	Representation of the terrain surface, steepness, wetness and to represent other geomorphological parameters	m
Slope	Slope inclination		degree
SAVI	Soil adjusted vegetation index		–
TWI	SAGA topographic wetness index		–
Landforms	USGS topographical landform classes		–
AWC	Available water content	Legacy information on moisture content	%
BD	Bulk density	restriction to root growth, infiltration, percolation and the ability of roots to reach moist zones in the soil	Mg m^−3^
Bedrck	Depth to bedrock	Depth to impenetrable layer	cm
Clay	Clay content of the soil	Legacy information on clay content	%
Sand	Sand content of the soil	Legacy information on sand content	%
Silt	Silt content of the soil	Legacy information on silt content	%
Drainage	FAO soil drainage classes	–	–
Geology	Geological formation	–	–
Lithology	FAO lithological classes	–	–
Riverdist	River distance		km
Temp	Spatial pattern of temperature	–	^o^C
Precip	Spatial pattern of precipitation	–	mm
Sent1A_VH	Calibrated sigmaO Sentinel-1A radar backscatter coefficients in VH polarization	Proxy for SM	dB
Sent1A_VV	Calibrated sigmaO Sentinel-1A radar backscatter coefficients in VV polarization	Proxy for SM	dB
Soil_types	Mapping units at series level	–	–
WatBal	Water balance	–	%
WatCov	Spatial coverage of hydrology networks	–	ha

### Selecting bandwidth for the GWPCA

Selecting an optimal bandwidth was the key to achieve an optimized GWPCA algorithm. Following the approach of Harris et al. [6], we adopted an automatic bandwidth selection process. The stack of auxiliary datasets had dimensions of 315, 579 and 21 (number of rows, columns and layers respectively) and 182,385 pixels. It was thus impossible within a reasonable time-span to automatically select a bandwidth using the entire auxiliary datasets, which had GWPCA loadings at each 182,385 pixel sites. This challenge was related to the required computing power and processing time in the cross validation algorithm, because each observation omitted was reconstructed using the principal components (PC) derived from the observations of the entire stack of auxiliary datasets. Therefore, we randomly selected 10% of the auxiliary datasets for use in the automatic bandwidth selection process. The selection of 10% of the datasets was guided by a series of cross validation evaluations. We identified an adaptive bandwidth of 16.2 km at *k =* 10 (principal components) as the optimum minimized fit between the score and auxiliary data.

### Development of the cost layer and the cost-constrained cLHC algorithm

**Fig. 4:**
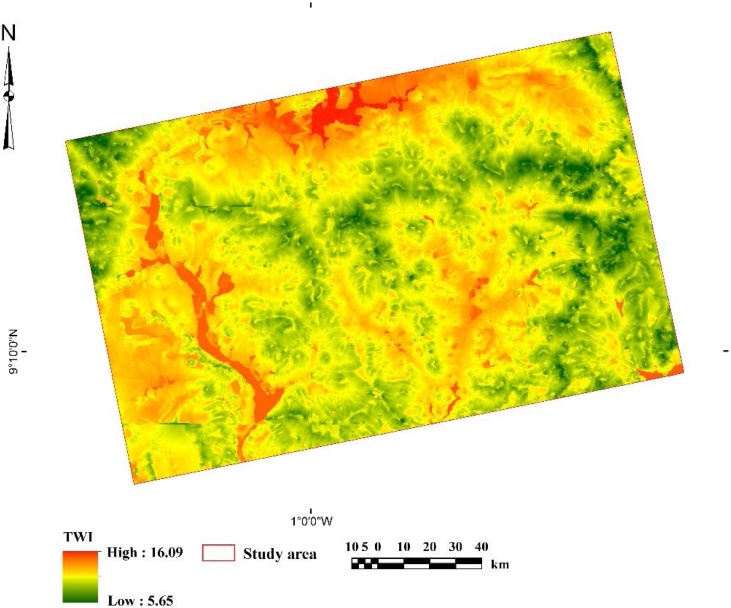
SAGA topographic wetness index (TWI) layer assigned as cost in the cLHC, simulating annealing at cooling temperature at iteration *j.*

In the cLHC annealing simulation process, model optimization was implemented by executing 1*10^4^–5*10^4^ iterations (increments of 1*10^4^). Because the user can define the number of cLHC selection outputs, we defined 38 locations in this study, as this was the maximum number of access tubes we had for the SM measurements. Thus, depending on the objectives and resources available for a particular study, users of this methodology can assign any maximum number of outputs. Roudier et al. [15] used rough terrain, surface gradient and distance to road or trail network as criteria to design their cost constraint in simulating the annealing process. In this study, we used a similar approach but a different key criterion, namely the topographic wetness index (TWI), as a suitable constraint indicator directly affecting SM. The TWI is generated with an upslope contribution area [22] and accounts for water redistribution within crest, ridges and depressions in an area [23]. The TWI gives an indication of the potential SM contribution areas within the top and bottom soil layers [24] and quantifies the spatial scale effects on hydrological processes [[25], [26], [27]]. In the TWI, slope inclination is classified at very short ranges to account even for slight changes in topography and local slope. In this study, low values represented crests and ridges, whereas high values represented depressions. We assumed that rainfall, infiltration, percolation and flooding by rivers during the rainy season are the only means by which SM is replenished in the soil layers of the study area. This assumption is based on the fact that in the study area, there is an inherent strong plinthic to petroplinthic horizon at ˜60–100 cm depth, which largely hinders contribution of ground water to SM [16,17,28]. Hence, we chose the TWI as cost-constraint criterion for assessing SM in the study area in the cLHC annealing simulation (Fig. 4). Using the *rsaga* package [13], we developed a TWI layer at a 100 m resolution.

## Method implementation and outputs

### GWPCA

**Fig. 5:**
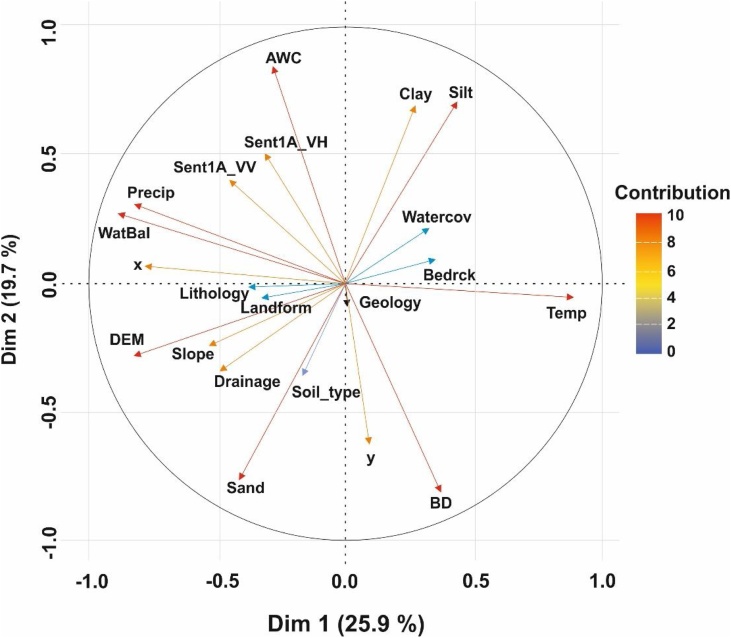
Correlation of the variation of list of auxiliary datasets in the feature space used in the GWPCA. AWC = available water content, BD = bulk density, Clay and Silt = clay and silt contents respectively, Sent1A_VV and Sent1A_VH = calibrated Sentinel-1 radar backscatter coefficient in VV and VH polarization respectively, Precip = precipitation, Bedrck = depth to bedrock, DEM = digital elevation model, WatBal = water balance, Watercov = water cover, Soil_type = soil mapping units and Temp = temperature.

Only PCs that accounted for eigenvalues ≥ 1 were considered. Temperature showed the strongest positive correlation, followed by bulk density, silt and clay contents (Fig. 5). Increasingly negative correlations were found for water balance < precipitation < DEM < drainage < sand content < available water content and sentinel-1 A. Within the rotational matrix of the global weighted PCs, available water content always showed the largest contribution in the list of auxiliary datasets, whereas sand content contributed least. Thus, we conclude that most of the variation was explained by available water content.

PC1 to PC6 together explained 91.5% of the observed variance (Supplementary material (SUPP) Table S1). Generally, the highest positive loading of 0.552 was recorded for PC5 and the lowest negative loading of -0.453 was recorded for PC6. Together, PC1 and PC2 accounted for 74.7% of the local variation in the list of auxiliary dataset used. These findings suggest that AWC, clay and silt content are the key factors that need to be considered to explain local variability of SM. By adding PC3, the explained variability reached 82.1%. PC4 explained an additional 3.5% of the observed local variation. In PC4, soil type showed the highest positive loading of 0.446, whereas river distance exhibited the lowest negative loading of -0.445. We thus conclude that the local soil types, affected by their distance to rivers, markedly influence the spatial variability of SM. Adding PC5 and PC6 resulted in a cumulative explained variability of 88.1% and 91.5%, respectively. River distance exhibited the lowest negative loadings in PC4, PC5 and PC6. AWC showed maximum positive loadings in the GWPCA two times. Thus, among the list of auxiliary datasets used in this study, AWC and river distance showed the strongest influence on the spatial variability of SM. The reason for the important role of distance to a river that we found in this case may be the dense river network and the generally low elevation within the area that leads to a far-reaching riparian influence.

While the PCs showed the observed variances in the auxiliary datasets, it also indicated the collinearity between the auxiliary datasets (SUPP Table S1). The larger or smaller the first or last PC, respectively, the stronger the collinearity between the auxiliary datasets [5]. Hence, local variables that caused the local collinearity in the auxiliary datasets was identified and eliminated via the multivariate glyph cartogram plot prior to its use in the cLHC [5]. The local spatial variability was analysed in the GWPCA using a 16.2 km bandwidth window around each data point in the auxiliary datasets. This bandwidth is equivalent to half of the maximum distance from river networks (minimum = 0 km, maximum = 32.3 km). The selected automatic bandwidth interprets as ˜50% of the entire auxiliary datasets were retained each time in the cross validation algorithm to calibrate the GWPCA. Thus ˜50% of the auxiliary datasets were used each time to explain the localised spatial variabilities of the study area. In addition, the automatic bandwidth ensured a balance between the local variation and locations with less influence on the spatial variability, as reported by Kumar et al. [7]. Our findings suggest that the use of GWPCA will account for the local influences and collinearity of each auxiliary datasets on the proposed SM measurements with regards to their locality. The final output of the cLHC ensures that the proposed sampling locations cover 91.5% of the locally occurring site conditions, represented by the auxiliary datasets.

### The cost-constrained cLHC algorithm

**Fig. 6:**
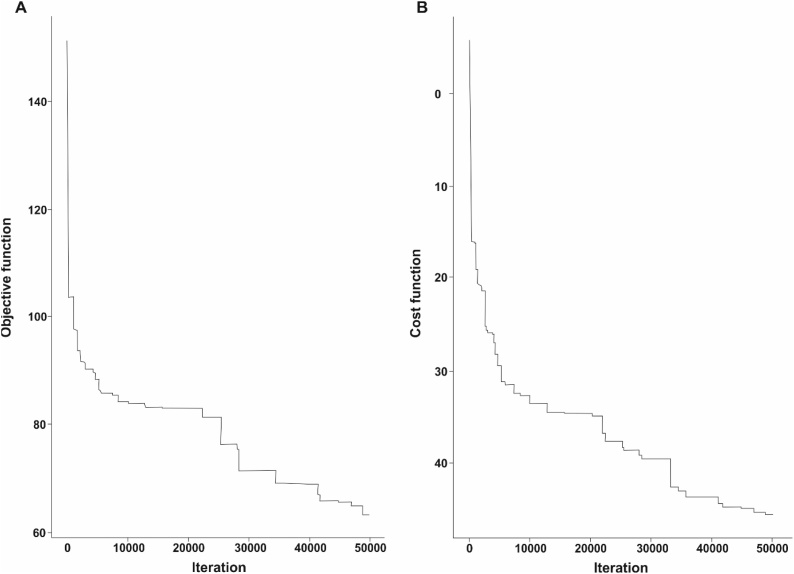
Evolution of the objective (A) and cost function (B) for the cLHC simulation from 1*10^4^–5*10^4^ iterations.

To implement the cLHC, the algorithm analyzed the selected PCs of the GWPCA to identify points in the landscape representing a Latin hypercube, similar to the approach of Minasny and McBratney [2]. In the subsequent series of iterations of this spatial representation of site conditions, the level of optimization of the objective function of the cLHC was shown at each iteration. Perturbations were recorded in both the objective and the cost-function optimization processes at <5*10^3^ iterations, similar to what Roudier et al. [15] reported. Contrary to the findings of Roudier et al [15], our results showed a clear full model optimization after 5*10^4^ iterations, both in the evolution and in the cost functions of the cLHC algorithm (Fig. 6). Between 1*10^4^ and 22*10^3^ iterations of the objective function, the optimization steadily increased until 5*10^4^ iterations were completed.

A comparison of the subsamples drawn from the selected PCs of the GWPCA used in the cLHC simulating annealing process is presented in SUPP Figure S1. Generally, the subsamples were within the first and third quartiles of the selected PCs of the auxiliary datasets. Only very small subsamples were drawn at locations with low TWI (ridges and crests). At sites with high TWI (depressions), large subsamples were drawn for use in the cLHC simulating annealing process. The reason for this difference is that the cLHC simulating annealing process suppressed sampling of locations with little or no influence on spatial SM variability in the study area (in this case, from ridges and crests). Thus, the probability of model selected sampling locations on ridges and crests was low.

**Fig. 7:**
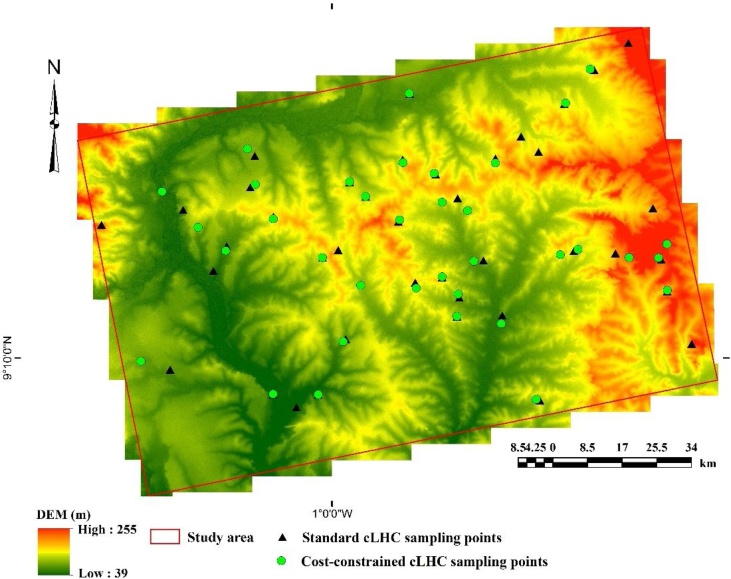
Single realization of the cLHC algorithm overlaid on a five-point *z* exaggerated digital elevation model. Green circles represent the cost-constrained cLHC and black triangles represent unconstrained cLHC.

A single realization of the cLHC output shows that the cost layer in the cLHC influenced the selection of sampling locations (Fig. 7). The cost-constrained cLHC stratified the selected sampling locations, based on the influence of TWI (similar to results reported by Levi and Rasmussen [4]). In our study, the selected sampling locations represented the soil property of interest and in the geographical space, as also reported by Hengl et al. [1]. Locations selected by unconstrained cLHC algorithms were dispersed across the entire study area (Fig. 7). The reason is that the cLHC optimization process accounted for all key landscape heterogeneities that occurred within the study area. The wide spatial distribution of sampling locations indicates that the cLHC effectively selected sampling locations, in terms of both the soil property of interest and the geographic space (Fig. 7). However, some locations were selected similarly under both the unconstrained and the cost-constrained cLHC algorithms. This hybrid approach enables a scientist to assess the local variability of a soil property of interest and to derive adequate sampling designs for analysing that soil property across a defined study area.

### Accuracy assessment

The highest (1) and lowest (0.3) average scores were achieved by 71% and 2.63%, respectively, of the model selected sampling locations. Test of the method showed a RMSE of 0.18 m with a correlation coefficient (R^2^) between actual and model selected location of 0.90. Our findings indicates that the model selected sampling locations were very close to the *in-situ* sampling locations. AT14 and AT21 were found not suitable probably due to the main input dataset in the GWPCA, which affected the output of the cLHC algorithm. Although 91.5% of the observed local variability in the auxiliary dataset was explained by the GWPCA, it must be emphasized that incorrect input parameters can limit the performance of the cLHC.

## Conclusion

In this paper, we have presented a new approach for selecting soil sampling locations that adequately represent both the soil property of interest and the geographical space across a study area. We implemented the proposed hybrid approach under a cost-constrained conditioned Latin hypercube algorithm, by using inputs from a global weighted principal component analysis. This method defines the local structure and accounts for localized spatial autocorrelation in explaining soil-landscape variability. The method also suppresses the occurrence of model-selected sampling locations in areas that are less representative of the soil property of interest. In addition, the method provide an appropriate base for selecting adequate sites for a given number of possible measurements. The proposed approach can guide the selection of adequate sites for soil measurements and installations of soil-monitoring stations, in the context of scientific studies and agricultural interventions.

## Conflict of interest

Authors declare no conflict of interest.

## References

[1] Hengl T., Rossiter D.G., Stein A. (2003). Soil sampling strategies for spatial prediction by correlation with auxiliary maps. Soil Res..

[2] Minasny B., McBratney A.B. (2006). A conditioned Latin hypercube method for sampling in the presence of ancillary information. Comput. Geosci..

[3] Biswas A., Zhang Y. (2018). Sampling designs for validating digital soil maps: a review. Pedosphere.

[4] Levi M.R., Rasmussen C. (2014). Covariate selection with iterative principal component analysis for predicting physical soil properties. Geoderma.

[5] Harris P., Brunsdon C., Charlton M. (2011). Geographically weighted principal components analysis. Int. J. Geogr. Inf. Sci..

[6] Harris P., Clarke A., Juggins S., Brunsdon C., Charlton M. (2015). Enhancements to a geographically weighted principal component analysis in the context of an application to an environmental data set. Geogr. Anal..

[7] Kumar S., Lal R., Lloyd C.D. (2012). Assessing spatial variability in soil characteristics with geographically weighted principal components analysis. Comput. Geosci..

[8] Comber A.J., Harris P., Tsutsumida N. (2016). Improving land cover classification using input variables derived from a geographically weighted principal components analysis. ISPRS J. Photogramm. Remote Sens..

[9] Charlton M., Brunsdon C., Demsar U., Harris P., Fotheringham S. (2010). Principal Components Analysis: From Global to Local.

[10] McBratney A.B., Santos M.M., Minasny B. (2003). On digital soil mapping. Geoderma.

[11] R Core Team (2017). A Language and Environment for Statistical Computing.

[12] Kassambara A., Mundt F. (2016). Package ‘factoextra’. Extract and Visualize the Results of Multivariate Data Analyses.

[13] Brenning A., Bangs D., Becker M., Schratz P., Polakowski F. (2018). Package ‘RSAGA’.

[14] Revelle W. (2018). Psych Package [Program].

[15] Roudier P., Beaudette D.E., Hewitt A.E. (2012). A conditioned Latin hypercube sampling algorithm incorporating operational constraints. Digital Soil Assessments and Beyond.

[16] Adu S.V. (1995). Soils of the Nasia River Basin, Northern Region, Ghana.

[17] Adu S.V., Asiamah R.D. (2003). Soils of the Yapei – Sawla Road Area, Northern Region, Ghana.

[18] IUSS Working Group WRB (2015). World Reference Base for Soil Resources.

[19] Junner N.R. (1940). Geology of the gold coat and Western Togoland. Bull. Gold Coast Geol. Surv..

[20] Mitchell T., Developers G. (2014). Geospatial Power Tools: GDAL Raster & Vector Commands. https://books.google.de/books?id=xQXqrQEACAAJ.

[21] Conrad O. (2006). SAGA. Aufbau, Funktionsweise und Anwendung eines Systems für geowissenschaftiche Analysen.

[22] Western A.W., Grayson R.B., Blöschl G., Willgoose G.R., McMahon T.A. (1999). Observed spatial organization of soil moisture and its relation to terrain indices. Water Resour. Res..

[23] Ballerine C. (2017). Topographic Wetness Index Urban Flooding Awareness Act Action Support, Will & DuPage Counties.

[24] Huang X., Shi Z.H., Zhu H.D., Zhang H.Y., Ai L., Yin W. (2016). Soil moisture dynamics within soil profiles and associated environmental controls. Catena.

[25] Grabs T., Seibert J., Bishop K., Laudon H. (2009). Modeling spatial patterns of saturated areas: a comparison of the topographic wetness index and a dynamic distributed model. J. Hydrol..

[26] Hofmeister L., Nave L., Drevnick P.E., Walter M.T. (2016). Topographic wetness indices, soil moisture, and water table dynamics identify hydrologic flow paths in a forest watershed. AGU Fall Meeting Abstracts.

[27] Lei S., Chen H., Bian Z., Liu Z. (2016). Evaluation of integrating topographic wetness index with backscattering coefficient of TerraSAR-X image for soil moisture estimation in a mountainous region. Ecol. Indic..

[28] Asiamah R.D., Dedzoe C.D. (1999). Plinthization—a threat to agricultural production. Ghana J. Agric. Sci..

