# Supplementary material for: A new method for selecting sites for soil sampling, coupling global weighted principal component analysis and a cost-constrained conditioned Latin hypercube algorithm
Source: MethodsX. 2019 Feb 8;6:284–99. doi: 10.1016/j.mex.2019.02.005 (PMC6377390; doi:10.1016/j.mex.2019.02.005)
Supplement: Supplementary file 1 [file mmc1.docx]

**LIST OF TABLES**

Table S1. Principal component matrix of the GWPCA

|  | **PC1** | **PC2** | **PC3** | **PC4** | **PC5** | **PC6** |
| --- | --- | --- | --- | --- | --- | --- |
| **EV** | 5.249 | 3.916 | 2.079 | 1.598 | 1.325 | 1.117 |
| **CPoV** | 0.747 | | 0.812 | 0.847 | 0.881 | 0.915 |
| **Loadings** | | | | | | |
| AWC | -0.150 | **0.382** | -0.177 | -0.118 | -0.079 | **0.259** |
| BD | 0.145 | **-0.417** | 0.141 | -0.006 | -0.026 | -0.197 |
| Bedrck | 0.175 | 0.040 | 0.289 | 0.295 | -0.031 | -0.261 |
| Clay | 0.179 | 0.365 | 0.162 | -0.002 | 0.270 | -0.102 |
| DEM | **-0.368** | -0.081 | 0.214 | -0.017 | 0.035 | -0.144 |
| Drainage | -0.257 | -0.150 | -0.229 | -0.146 | 0.083 | -0.129 |
| Geology | -0.025 | -0.051 | -0.326 | 0.046 | 0.027 | -0.077 |
| Landforms | -0.168 | -0.004 | -0.140 | -0.107 | **0.552** | -0.147 |
| Lithology | -0.168 | 0.056 | **0.297** | 0.008 | 0.176 | -0.408 |
| Precip | -0.325 | 0.181 | 0.266 | 0.077 | -0.092 | 0.224 |
| Riverdist | -0.129 | 0.024 | 0.059 | **-0.445** | **-0.187** | **-0.453** |
| Sand | -0.220 | -0.388 | -0.118 | 0.138 | -0.152 | 0.034 |
| Sent1A_VH | -0.150 | 0.261 | **-0.355** | 0.317 | -0.141 | -0.264 |
| Sent1A_VV | -0.197 | 0.213 | -0.339 | 0.335 | -0.160 | -0.254 |
| Silt | 0.216 | 0.349 | 0.053 | -0.248 | 0.016 | 0.044 |
| Soil_types | -0.076 | -0.173 | 0.167 | **0.446** | 0.131 | 0.258 |
| Temp | **0.374** | -0.083 | -0.270 | 0.000 | -0.002 | 0.081 |
| WatBal | -0.345 | 0.172 | 0.249 | 0.067 | -0.090 | 0.199 |
| WATCov | 0.155 | 0.115 | 0.014 | 0.373 | 0.393 | -0.168 |
| Slope | -0.260 | -0.099 | -0.162 | -0.132 | 0.411 | 0.145 |
| **Maximum** | 0.374 | 0.382 | 0.297 | 0.446 | 0.552 | 0.259 |
| **Minimum** | -0.368 | -0.417 | -0.355 | -0.445 | -0.187 | -0.453 |

PC = principal component, EV = eigenvalues, CPoV = cumulative proportion of variation and bold values represents minimum and maximum values.

**LIST OF FIGURES**


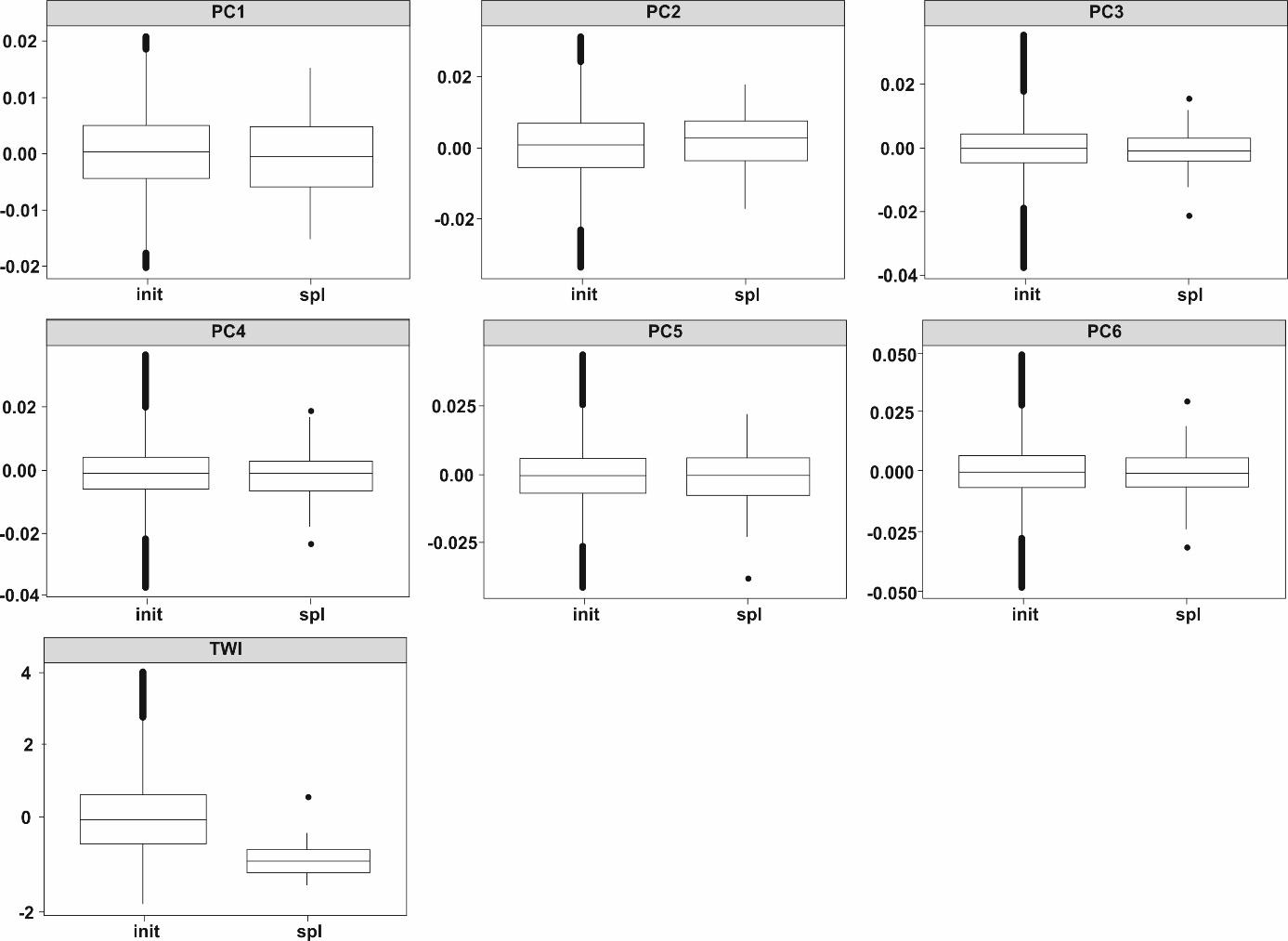


Figure S1. Distribution of subsamples (spl) drawn from the selected PCs of the GWPCA (init) used in the cLHC simulating annealing process. TWI = SAGA topographic wetness index.

**SOURCE CODE**

Source codes (R scripts) for full method implementation on GitHub repository (https://github.com/kanj241/PhD/blob/master/Sampling_desi-gn).
